# A Systematic Critical Appraisal of Clinical Practice Guidelines in Juvenile Idiopathic Arthritis Using the Appraisal of Guidelines for Research and Evaluation II (AGREE II) Instrument

**DOI:** 10.1371/journal.pone.0137180

**Published:** 2015-09-10

**Authors:** Christine A. M. Smith, Karine Toupin-April, Jeffrey W. Jutai, Ciarán M. Duffy, Prinon Rahman, Sabrina Cavallo, Lucie Brosseau

**Affiliations:** 1 School of Epidemiology, Public Health and Preventive Medicine, University of Ottawa, Ottawa, Ontario, Canada; 2 Children’s Hospital of Eastern Ontario Research Institute, Ottawa, Ontario, Canada; 3 Department of Pediatrics, Faculty of Medicine, University of Ottawa, Ottawa, Ontario, Canada; 4 Interdisciplinary School of Health Sciences, University of Ottawa, Ottawa, Ontario, Canada; 5 Department of Pediatrics, Children’s Hospital of Eastern Ontario, Ottawa, Ontario, Canada; 6 Department of Community Health and Epidemiology, Dalhousie University, Halifax, Nova Scotia, Canada; 7 École de Santé Publique, Université de Montréal, Montréal, Québec, Canada; 8 School of Rehabilitation Sciences, Faculty of Health Sciences, University of Ottawa, Ottawa, Ontario, Canada; VU University Medical Center, NETHERLANDS

## Abstract

**Objectives:**

The objectives of this review are to: 1) appraise the methodological quality of clinical practice guidelines (CPGs) in juvenile idiopathic arthritis (JIA) providing pharmacological and/or non-pharmacological intervention recommendations, and 2) summarize the recommendations provided by the included CPGs and compare them where possible.

**Methods:**

A systematic search was performed. Three trained appraisers independently evaluated the methodological quality of the CPGs using a validated and reliable instrument, the Appraisal of Guidelines in Research and Evaluation II. Six domains were considered: 1) score and purpose; 2) stakeholder involvement; 3) rigor of development; 4) clarity of presentation; 5) applicability; and 6) editorial independence. The domains consist of a total of 23 items each scored on a 7-point scale. High quality CPGs were identified if they had a domain score above 60% in rigor of development, and two other domains.

**Results:**

Of the three included CPGs, the Royal Australian College of General Practitioners (RACGP) and American College of Rheumatology (ACR) CPGs were considered to be of high quality, but the German Society for Pediatric Rheumatology was of lower quality. Domains one to four had high domain scores across the guidelines (mean (standard deviation)): 72.76 (13.80); 66.67 (9.81); 64.67 (7.77); and 87.00 (9.64), respectively. Lower scores were obtained for applicability (14.00 (5.57)) and editorial independence (43.44 (7.02)). Recommendations varied across CPGs due to differences in context, target audience (general practitioners, rheumatologists, and other multidisciplinary healthcare professionals) and patients’ disease presentations. Despite this variability, progression of pharmacological treatment did not conflict between CPGs. Recommendations for non-pharmacological interventions were vague and the interventions considered varied between CPGs.

**Conclusions:**

Overall, recommendations were based on a paucity of evidence and weak study designs. Further research is needed on interventions in JIA, as well as higher quality CPGs to facilitate implementation of the best evidence-based recommendations in clinical practice.

## Introduction

Juvenile idiopathic arthritis (JIA) is the most common rheumatic disease of childhood, with the precise etiology unknown and an incidence of 1 in 10,000 in children under 16 years of age [1,2]. A diagnosis requires that arthritis be present for a minimum of six weeks in patients younger than 16 years old [3,4]. There are seven onset types of JIA: systemic arthritis, oligoarthritis, polyarthritis (rheumatoid factor negative), polyarthritis (rheumatoid factor positive), psoriatic arthritis, enthesitis related arthritis, and undifferentiated arthritis [3,4]. These categories are mutually exclusive and differ based on the number of joints affected by arthritis (4 or fewer joints for oligoarthritis and 5 or more joints for polyarthritis), the presence of serological markers (e.g. rheumatoid factor positive or negative polyarthritis), or the area of the body affected (e.g. tenderness of the sacroiliac joint in enthesitis related arthritis). Symptoms of JIA include joint symptoms such as joint pain, swelling, and stiffness, for all onset types but also systemic symptoms such as fever and rash for those with systemic arthritis [2,5–8].

While most children do very well overall with this condition, an important proportion exhibit reduced quality of life, often as a consequence of associated joint damage with resulting pain which ultimately leads to a reduction in their ability to complete daily tasks and participate in activities [7,9]. Early pharmacological and non-pharmacological treatment of the disease is imperative for the prevention of irreversible soft tissue and joint damage [10]. Pharmacological interventions for JIA include non-steroidal anti-inflammatory drugs (NSAIDs), disease-modifying antirheumatic drugs (DMARDs), biologics, and glucocorticoids (GCs) (both as systemic treatment and now more commonly through intra-articular injection). Most treatments can be used to control and delay the progression of symptoms of JIA, as well as prevent joint damage over the long term [11]. Non-pharmacological interventions, such as physiotherapy interventions (e.g. therapeutic exercises, massage), coupled with orthotics when required, may help patients maintain their joint range of motion and functional status while also contributing to maintenance of an increase in bone mineral density, and ultimately to the prevention of osteopenia [12,13]. This combined multi-disciplinary approach to care is essential for overall better management of symptoms and leads to better ultimate outcomes [14,15].

Clinical practice guidelines (CPGs) are defined as “systematically developed statements to assist practitioner and patient decisions about appropriate healthcare for specific clinical circumstances” (p. 38) [16]. To the authors’ knowledge, there are no publications that have appraised the methodological quality of existing pharmacological and/or non-pharmacological CPGs for JIA using the Appraisal of Guidelines in Research and Evaluation (AGREE) or AGREE II [17] instruments.

The primary objective of this systematic review is to evaluate the methodological quality of CPGs providing specific recommendations for pharmacological and/or non-pharmacological interventions for patients with JIA. A secondary aim is to summarize the content of the CPG recommendations and to compare recommendations across CPGs where possible.

## Materials and Methods

The systematic review of CPGs used the Cochrane Methodology [18] to identify, select and analyze the data and the Preferred Reporting Items for Systematic Reviews and Meta-Analyses statement to guide the reporting of the systematic review (S1 Appendix). Ethics approval was not required, as this work was based on a systematic literature review.

### Systematic review of the literature

This systematic review used a similar methodology to previous studies in adult rheumatology [19,20]. A systematic search of the literature was performed in Medline (Ovid), the Cumulative Index to Nursing and Allied Health Literature, Embase (Ovid), and PubMed with the assistance of a librarian specialized in rehabilitation sciences (ML). The search limits included CPGs published in English between January 2003 and December 2014. Additionally, a hand search was performed in June 2015 in the guideline inventories Canadian Medical Association Infobase, Physiotherapy Evidence Database and National Guideline Clearinghouse, in the reference lists of included CPGs, and on the following websites of known rheumatology groups who develop CPGs: American College of Rheumatology (ACR) (http://www.rheumatology.org/), Royal Australian College of General Practitioners (RACGP) (http://www.racgp.org.au/), the Canadian Rheumatology Association (http://rheum.ca/), the European League Against Rheumatism (http://www.eular.org/), National Institute for Health and Care Excellence (http://www.nice.org.uk/), Scottish Intercollegiate Guidelines Network (http://www.sign.ac.uk/), and the British Society for Rheumatology (http://www.rheumatology.org.uk/). Pediatric rheumatology experts were also consulted on the presence of other existing CPGs. S2 Appendix contains the full search strategies with key words.

#### Selection criteria

S3 Appendix contains the full details of the selection criteria and S4 Appendix provides the reasons for exclusion of certain citations.

### Instruments

The AGREE II instrument assesses the methodological quality of CPGs in six main areas (domains): 1) scope and purpose; 2) stakeholder involvement; 3) rigor of development; 4) clarity of presentation; 5) applicability; and 6) editorial independence. Each domain is divided into smaller categories called items, with a minimum of 2 items (domain 6) and a maximum of 8 items (domain 3), for a total of 23 items [21].

### Assessment of CPG quality

#### Training the appraisers

Three trained individuals (CS, SR, KTA) formed two pairs of appraisers and received the same training with the AGREE II online tutorial [22]. Prior to evaluating the JIA CPGs, the appraisers reviewed a CPG on osteoarthritis using the AGREE II instrument to familiarize themselves with the instrument. Each appraiser independently scored the CPGs using the AGREE II items and then assigned an overall quality rating to the CPG using the same 7-point scale. For each CPG, appraisers also indicated whether they: recommended its use by clinicians for practice, recommended its use with some modifications, or did not recommend its use.

#### Grading of the items and domains

The AGREE II user manual was used by appraisers to refer to the criteria for each item [21]. A grade was assigned for each item using a 7-point scale indicating the level of agreement with each statement about the methodological quality of the CPGs. A score of 1 was given if none of the criteria for an item were met or the item was reported very poorly, while a score of 7 was given for an item if it met all the criteria and was well reported. The domain score was calculated using the sum of the item scores in a given domain and transforming the number into a percentage of the maximum score that domain could obtain. For this review, a high quality CPG required a score of 60% for rigor of development (domain 3) as well as 60% in any two other domains [23–25].

#### Calculating the domain scores

After all appraisers finished grading the CPGs, their scores were entered into an AGREE II score calculator developed by the Capacity Enhancement Program at McMaster University [26]. An overall methodological quality score of all items in all domains was computed on a 7-point scale for each CPG. The concordance calculator was used to ensure the correct calculation of domain scores ($\bar{\text{x}}$ (SD)) from the equation provided in the AGREE II user’s manual [21].

#### Discrepancies in rater scores

If the concordance calculator computed a medium or high discrepancy in domain scoring among the three appraisers, a fourth appraiser (LB) resolved the discrepancies. A medium discrepancy was defined as individual appraisers’ scores for a domain being 1.5 to 2 standard deviations from the mean domain score. A high discrepancy was greater than 2 standard deviations from the mean domain score [26]. To reduce the level of error, the three appraisers independently reviewed for a second time all items where there were medium discrepancies or where scores differed by three or more points between at least two appraisers.

#### Inter-rater reliability

Inter-rater reliability was examined by comparing the individual item scores of each appraiser and ensuring there was only a low discrepancy (less than 1.5 standard deviations from the mean domain score) with the concordance calculator [26].

#### Summary of CPG recommendations

The recommendations provided by each CPG were presented based on types of patients (e.g. clinical scenarios or JIA onset types), rather than by intervention to take into consideration the complexity of care for patients with JIA. Any treatment algorithms presented by the CPGs were also summarized to indicate the progression of treatment for JIA that the CPGs recommended.

## Results

### Search results

The systematic review provided a total of 90 citations and one citation was found through a hand search (S1 Fig). There were 42 citations in Embase, 20 in the Cumulative Index to Nursing and Allied Health Literature, 2 in Medline, 11 in PubMed, 1 in the Canadian Medical Association Infobase, 1 in the Physiotherapy Evidence database, and 13 in National Guidelines Clearinghouse. After an initial screening process reviewing titles and/or abstracts, 11 CPGs had their full text examined. Three CPGs met the inclusion criteria and were evaluated using the AGREE II instrument. These were the German Society for Pediatric Rheumatology (GKJR) [27], RACGP [28] and ACR [29,30] CPGs. ACR published a partial update in 2013 [30] for new pharmacological therapies to treat systemic JIA (sJIA) and it used the same methodology as ACR in 2011 [29], thus it was treated as an extension to the main CPG rather than providing a separate AGREE II score. There were no additional CPGs found in the updated hand search or through consultation of pediatric rheumatology experts. S1 Fig contains a Preferred Reporting Items for Systematic Reviews and Meta-Analyses (PRISMA) flow diagram of the systematic literature search and S4 Appendix indicates the excluded citations and the rationale behind their exclusion.

### Methodological quality of CPGs

The overall methodological quality score for each CPG was (mean (standard deviation)): 5.67 (0.58) for RACGP [28]; 4.33 (0.58) for ACR [29,30]; and 3.67 (0.58) for GKJR [27]. All three CPGs obtained scores greater than 60% in domains 2 (stakeholder involvement) and 4 (clarity of presentation). However, none of the CPGs reached a score of 60% for domains 5 (applicability) and 6 (editorial independence). Of the three included CPGs, both ACR [29,30]; and RACGP [28] were considered high quality because they obtained scores greater than 60% in four of six domains, including rigor of development (domain 3). GKJR [27] had two domain scores above 60%, but received a result slightly below the threshold of 60% (with a score of 56%) for rigor of development. While one of the appraisers said they would not recommend GKJR [27], the other two said that they would recommend it with modifications. Therefore, the recommendations were still considered because its score was close to the threshold. However, healthcare professionals should use their discretion when applying recommendations from GKJR [27]. Table 1 provides the scores that all CPGs received in each domain and an overall quality rating of the CPGs.

**Table 1:** Quality scores of included CPGs using AGREE II.

AGREE II Domains	ACR [27,29,30]	GKJR [27]	RACGP [28]	$\bar{\text{x}}$ (SD) (%)	Range of quality scores (%)
Domain 1. Scope and purpose (%)	78%	57%	83%	72.67 (13.80)	26
Domain 2. Stakeholder involvement (%)	61%	61%	78%	66.67 (9.81)	17
Domain 3. Rigor of development (%)	67%	56%	71%	64.67 (7.77)	15
Domain 4. Clarity of presentation (%)	91%	76%	94%	87.00 (9.64)	18
Domain 5. Applicability (%)	8%	15%	19%	14.00 (5.57)	11
Domain 6. Editorial independence (%)	50%	36%	44%	43.33 (7.02)	14
Overall quality rating of the CPG^a^ (AGREE II scoring system: 1–7) ($\bar{\text{x}}$(SD))	4.33 (0.58)	3.67 (0.58)	5.67 (0.58)		

ACR: American College of Rheumatology; GKJR: German Society for Pediatric Rheumatology; RACGP: Royal Australian College of General Practitioners; $\bar{\text{x}}$: mean; SD: standard deviation.

^a^Rating established using the AGREE II 7-point scale.

#### Domain 1: Scope and purpose

The CPGs had scores above 60% for scope and purpose except for GKJR [27] which had a score of 57%. The main reason for the score below 60% was that there was no detailed search strategy provided and this made the health questions covered by the CPG unclear. The overall objectives of the CPG and the population to whom the CPG applied were in general well described for all CPGs.

#### Domain 2: Stakeholder involvement

All three CPGs scored above 60%, although the mean quality score was lower than domain 1. This is largely due to unclear descriptions of how the CPGs sought the views and preferences of the target population. A description of the CPG development group was acceptable, though there was not always a description of disciplines or individual roles of those involved in the included CPGs.

#### Domain 3: Rigor of development

ACR [29,30] and RACGP [28] were very similar in their quality scores, which were both above 60%. GKJR [27] received a lower score of 56%. The main reason for the borderline score was there was no procedure given for updating the CPG, which led to scores of 1 and 2. The other two CPGs still had moderate scores for item 14 because the information provided was somewhat limited. In general, the CPGs used systematic methods in searching for evidence, as well as provided an obvious link between recommendations and their evidence.

#### Domain 4: Clarity of presentation

This domain received high scores from all three CPGs and the highest mean quality score (SD) was 87.00% (9.64%) (Table 1). Each CPG provided a type of treatment algorithm for pharmacological interventions and provided consensus statements for which interventions were recommended.

#### Domain 5: Applicability

Consistent across the CPGs, this domain had the lowest scores ranging from 8% to 19% (Table 1). All of the CPGs scored very poorly because they did not clearly state the costs and resources that were needed in order to implement the CPG, which was required by the AGREE II instrument [21,27–29]. Criteria for monitoring the implementation of recommendations and assessing the CPG’s impact were either not provided or simply involved referring readers to government documents [27].

#### Domain 6: Editorial independence

Of all the domains, editorial independence had the second lowest mean quality score at 43.33% (7.02%) (Table 1). While the funding body may have been mentioned, ACR and GKJR did not provide an explicit statement to declare that the funding body did not have an influence on the guideline content [27,29] and it was not entirely clear to the appraisers whether one of the members in the RACGP working group, who was an advisor for the funding source, influenced the CPG [28]. There was frequently missing information for recording the competing interests for members of the guideline development group in all CPGs.

#### Inter-rater reliability

The domain scores entered into the concordance calculator showed low discrepancy between pairs of assessors except for stakeholder involvement (domain 2), which required review for the ACR [29,30] and the GKJR [27] CPGs. The appraisers were asked to review their scores in domain 2 and determine if they had overlooked or misunderstood information provided in the CPGs when scoring the items. No change in the appraisers’ scores was made if they had originally found the relevant information and had understood it. Upon making these revisions, the medium discrepancy level (individual scores being between 1.5 and 2 standard deviations from the mean) for these two domains were resolved. In addition, each appraiser’s raw data scores for all 23 items were fairly consistent with only a few instances when two appraisers had scores varying by three points on the 7-point scale.

### Summary of recommended interventions

The three CPGs were developed for different target audiences, presented recommendations for varying clinical presentations of the disease, and used different grading systems that incorporated the strength of recommendations provided and also the level of evidence of the studies forming the recommendations. Table 2 describes these differences between the CPGs and S5 Appendix indicates the grading system used by each CPG. Many recommendations were based on a paucity of evidence, often relying on non-randomized controlled trials, observational studies, expert opinion, or extrapolations from adult studies. Summaries of the specific pharmacological and non-pharmacological recommendations provided by the CPGs are presented in Tables 3 and 4, respectively. Please note that these represent summaries only and readers should consult the original CPGs prior to treating patients with JIA.

**Table 2:** Scope and context of the CPGs.

	ACR [27,29,30]	GKJR [27]	RACGP [28]
Population of patients with JIA	History of arthritis in ≤ 4 active joints, history of arthritis in ≥ 5 active joints, systemic JIA (sJIA) with active systemic features (no active arthritis), sJIA with active arthritis (no systemic features), and sJIA with features concerning for macrophage activation syndrome (MAS)	Specific recommendations occasionally provided for oligoarthritis, polyarthritis, and sJIA onset types; otherwise the recommendations do not specify all onset types according to the International League of Associations for Rheumatology	Oligoarticular, polyarticular (RF negative), polyarticular (RF positive), systemic, enthesitis related, psoriatic and undifferentiated JIA onset types
Target audience	Pediatric rheumatologists	Physicians, affiliated professionals in healthcare, physical therapists, occupational therapists, and others caring for patients with JIA	General practitioners plus physical therapists, occupational therapists, sports medicine workers, podiatrists, dieticians, psychologists, pharmacists, nurses, community health workers
Range of pharmacological interventions	NSAIDs, glucocorticoids (injection and systemic), nonbiologic DMARDs, biologic DMARDs (sJIA)	NSAIDs, glucocorticoids (injection and systemic), prednisone/prednisolone, DMARDs, biologics	NSAIDs, topical NSAIDs, simple analgesics, weak opioids, complementary and alternative medicines
Range of non-pharmacological interventions	N/A	Electrotherapy, ultrasound, exercise, massage, lymph drainage, orthotics management (corrective appliances/splints), physiotherapy, occupational therapy, psychological support, socio-pedagogical care, synovectomy, thermotherapy	Complementary and alternative physical therapies, exercise (land-based and aquatic), nutritional therapy, orthotics management (corrective appliances/splints), thermotherapy
Notes	The 2013 update focused solely on sJIA; no discussion of contraindications, intolerance, tapering or discontinuing medications in the event of inactive disease	Excluded information on medication resistance or obscure and difficult arthritis cases	Main focus is on early diagnosis, initiation of intervention, and referral to pediatric rheumatologists; recommendations are for short term care and then planning and management for long-term care; multidisciplinary and developed for the Australian healthcare context

sJIA = systemic JIA; MAS = macrophage activation syndrome; NSAIDs = non-steroidal anti-inflammatory drugs; DMARDs = disease modifying antirheumatic drug

**Table 3:** Summary of recommendations for pharmacological interventions in JIA based on included CPGs. N.B. This is meant as a summary of the recommendations only. Please refer to the official CPGs before providing treatment to patients.

	ACR 2011 & 2013 [29,30]		GKJR 2012 [27]		RACGP 2009 [28]	
	Recommend: Yes/No/Unsure (Grade)	Details	Recommend: Yes/No/Unsure (Grade)	Details	Recommend: Yes/No/Unsure (Grade)	Details
**NSAIDs**						
Celecoxib			**Yes** (Grade B-C, level I)	not been approved in children; use if diclofenac, naproxen, ibuprofen and indomethacin are contraindicated		2–4 mg/kg b.i.d.
Diclofenac			**Yes** (Grade A, level I)	≥14 years old		1 mg/kg b.i.d.
Ibuprofen			**Yes** (Grade A, level I)	≥6 months; treat pain, fever		10 mg/kg 3–4 times daily
Indomethacin			**Yes** (Grade A, level II)	≥2 years old		0.5–1.0 2–3 times daily
Meloxicam			**No** (Level II)	≥15 years old; recommendation grade not given		0.15–0.30 mg/kg o.d.
Naproxen			**Yes** (Grade A, level I)	≥1 year old		5–7.5 mg/kg b.i.d.
Piroxicam						0.2–0.4 mg/kg o.d.
NSAID monotherapy	**1)** Hx ≤4 joints: **Yes** (Level B); **2)** Hx ≥5 joints: **Yes** (Level C); **3)** sJIA active systemic features (no active arthritis): **Unsure/No** (Level D); **4)** sJIA, active arthritis (no active systemic features): **Yes** (Level B)	**1)** with GC injections; for low disease activity (DA), no joint contracture, no features of poor prognosis (FPP); **2)** without GC injections uncertain if active arthritis; do not continue >2 months if active arthritis; **3)** appropriate before diagnosis and evaluating systemic arthritis, uncertain if active fever–inappropriate when fever and MD global ≥7/10; initial Tx if MD global <5 + any AJC; inappropriate if MD global ≥5 + AJC >0; inappropriate to continue Tx >1 month if DA continued; **4)** initiate if low DA without FPP (with/without GC injections); uncertain to continue >1 month for any DA level (Level D); if no previous Tx for 1 month max if AJC >0 (Level D); inappropriate for >2 months if continued disease activity (Level D)			**Yes** (Grade B)	Initial drug of choice to reduce pain and inflammation; see RACGP 2009 for details on dosing of specific NSAIDs
Topical NSAIDs					**No** (Grade D)	No studies available
**Steroids**						
Glucocorticoid (intra-articular injection)	**1)** Hx ≤4 joints: **Yes** (Level C); should show improvement for initial 4 months (Level A); **3)** sJIA active systemic features (no active arthritis): **Yes** (Level C); **4)** sJIA, active arthritis (no active systemic features): **Yes** (Level C)	**1)** any pt active arthritis, any disease level, prognostic features, or joint contracture; **3)** can consider adding it any time during treatment; **4)** initial Tx if AJC ≤4; uncertain as monotherapy if AJC >4; **unsure** about repeat injections as only Tx for ≥1 joint	**Yes** (Grade B, level I)	Triamcinolone hexacetonide; possible as part of first line Tx; triamcinolone hexacetonide preferred over triamcinolone acetonide due to efficacy		
Glucocorticoid (systemic)	**3)** sJIA active systemic features (no active arthritis): **Yes** (Level D); **5)** sJIA with features concerning for MAS: **Yes** (Level C)	**3)** initial Tx active fever and MD global ≥7; initiate after ≤2 wks for all pts with active fever (Level C); no published data on doses and routes; initial monotherapy (oral or IV) up to 2 weeks max when MD global <5 + AJC >4 OR MD global ≥5 (Level C); inappropriate to continue GC monotherapy ≥1 month if DA continues (Level D); continued disease Tx after NSAID monotherapy failed when MD global <5 + AJC >0 OR MD global ≥5 (Level C); can consider adding GC treatment any time (Level D); **5)** GC monotherapy ≥2 weeks is inappropriate when continued features concerning MAS (Level D)	**Yes** (Grade A, level III)	For highly AD; for pts with sJIA, complications (uveitis, pericardial effusion), RF-positive JIA; use while waiting for therapeutic effect of DMARD Tx to be complete; not recommended for long-term use; not recommended to continuously give 0.2 mg/kg of a prednisolone equivalent to pts		
Prednisolone/prednisone			**Yes** (Level III)	for severe sJIA; can be used as oral medium- or high-dose Tx; can also be used as IV pulse Tx; recommendation grade not given		
**DMARDs**						
Azathioprine			(Level II)	not been approved in children; 1.5–3 mg/kg per day orally in 1–2 doses		
Leflunomide	**1)** Hx ≤4 joints: **Unsure**; **2)** Hx ≥5 joints: Yes (Level B); **3)** sJIA active systemic features (no active arthritis): **Yes** (varies); **4)** sJIA, active arthritis (no active systemic features): **Yes** (Level C); **5)** sJIA with features concerning for MAS: **No** (Level D)	**2)** initial Tx when high DA and FPP; use after trial of NSAIDs for high DA and no FPP; **3)** for continued disease Tx if MD global <5 + AJC >0 after GC monotherapy (Level C), an IL-inhibitor (Level D), or tocilizumab (Level D); if MD global ≥5 + AJC >0 after trial of IL-1 inhibitor/tocilizumab (Level C); inappropriate if AJC = 0 + any MD global (Level D); **4)** initial Tx when AJC >4; continued disease Tx if AJC >0 after intraarticular injection (level C), NSAID monotherapy (Level C), an IL-1 inhibitor (Level D), or tocilizumab (Level D)	**Yes** (Grade B, level II)	Use only if MTX/etanercept insufficient at treating disease; not been approved in children	Not assessed	GPs to refer patient to pediatric rheumatology specialist
Methotrexate	**1)** Hx ≤4 joints: **Yes** (Level C); **2)** Hx ≥5 joints: **Yes** (Level B); **3)** sJIA active systemic features (no active arthritis): **No** (Level B); **4)** sJIA, active arthritis (no active systemic features): **Yes** (Level B); **5)** sJIA with features concerning for MAS: **No** (Level D)	**1)** Initial Tx if high DA and FPP; start after GC injections if moderate/high DA but no poor prognosis; **2)** Initial Tx if moderate/high DA and FPP; initial 1 month after NSAIDs when low DA and FPP; initiate 1–2 months after NSAIDs when moderate DA but no FPP; **3)** for initial management active fever without active arthritis; for continued disease Tx if MD global <5 + AJC >0 after GC monotherapy (Level C), an IL-inhibitor (Level D), or tocilizumab (Level D); if MD global ≥5 + AJC >0 after trial of IL-1 inhibitor/tocilizumab (Level C); inappropriate if AJC = 0 + any MD global (Level D); **4)** if active arthritis ≤1 month after NSAID monotherapy (with/without GC injections); initial Tx when AJC >4 (Level C); continued disease Tx if AJC >0 after intraarticular injection (level C), NSAID monotherapy (Level C), an IL-1 inhibitor (Level D), or tocilizumab (Level D)	**Yes** (Grade A, level I)	If NSAIDs and/or GC injections ineffective; use when continuously need systemic GCs OR if high DA; 10–15 mg/m^2^ oral/SC; ≥ 2 years old; for poly-JIA, psoriatic JIA, uveitis, collagenosis	Not assessed	GPs to refer patient to pediatric rheumatology specialist
Sulfasalazine	**1)** Hx ≤4 joints: **Yes** (Level B); **2)** Hx ≥5 joints: **Unsure**	**1)** after GC injection or NSAIDs with moderate/high DA; for enthesitis-related JIA; uncertain for other JIA onset types; **2)** for Tx initiation	**Yes** (Grade B, level II)	Use if MTX/etanercept insufficient	Not assessed	GPs to refer patient to pediatric rheumatology specialist
Nonbiologic DMARD combinations	**1)** Hx ≤4 joints: **Unsure**; 2) Hx ≥5 joints: **Unsure**; **3)** sJIA active systemic features (no active arthritis): **Unsure**; **4)** sJIA, active arthritis (no active systemic features): **Unsure**	**1)** MTX + sulfasalazine and/or hydroxychloroquine; **2)** MTX + sulfasalazine and/or hydroxychloroquine; **3)** MTX + leflunomide and/or a calcineurin inhibitor) for any AJC or MD global; **4)** initiation of a combination (MTX + leflunomide and/or a calcineurin inhibitor) at any AJC				
**Biologics**						
Abatacept	**1)** Hx ≤4 joints: **Unsure**; 2) Hx ≥5 joints: **Yes** (Level B); **3)** sJIA active systemic features (no active arthritis): **Yes** (Level D); **4)** sJIA, active arthritis (no active systemic features): **Yes** (Level B); **5)** sJIA with features concerning for MAS: **No** (Level D)	**1)** unsure before starting TNFα inhibitor; **2)** after 4 months TNFα inhibitor when moderate/high DA and FPP; start after receiving >1 TNFα inhibitor sequentially when moderate/severe DA with FPP; **3)** if MD global ≥5 + AJC >4 after trying an IL-1 inhibitor and tocilizumab sequentially; inappropriate when AJC = 0, except if pt tried IL-1 inhibitor and tocilizumab sequentially (unsure); inappropriate if MD global <5 + AJC >0 OR MD global ≥5 + AJC <4 (Level D); appropriate only if had IL-1 inhibitor and tocilizumab sequentially (Level D); unsure if pt tried DMARD + IL-1 inhibitor/tocilizumab; **4)** start if received MTX and TNFα inhibitor & has high DA OR moderate DA with poor prognosis; continued disease Tx if AJC >0 after MTX/leflunomide (Level B), anakinra (Level D), or tocilizumab (Level D); **5)** not for initiation	**Yes** (Grade C, level III)	≥ 6 years old; for pts with poly-JIA (non-systemic) when unresponsive MTX and TNFα inhibitors	Not assessed	GPs to refer patient to pediatric rheumatology specialist
Anakinra	**2)** Hx ≥5 joints: **Unsure**; **3)** sJIA active systemic features (no active arthritis): **Yes** (Level C); **4)** sJIA, active arthritis (no active systemic features): **Yes** (Level C); **5)** sJIA with features concerning for MAS: **Yes** (Level C)	**3)** start if active fever and FPP regardless of intervention currently taken; for all patients who still have/develop active fever with GCs; initial Tx if MD global <5 + AJC >4 OR if MD global ≥5; for continued disease Tx after GC monotherapy (Level A) OR NSAID monotherapy (Level C); **4)** start in pts who were given MTX and have moderate/high DA; if Tx with MTX + TNFα inhibitor OR MTX + abatacept in pts with moderate/high DA; inappropriate to initiate anakinra later in disease course–better earlier on; continued disease Tx if AJC >4 after GC injection OR NSAID monotherapy failed (Level B) OR if AJC >0 after Tx of MTX/leflunomide	**Yes** (Grade A, level II)	Not been approved in children; ≥ 2 years old; for refractory sJIA	Not assessed	GPs to refer patient to pediatric rheumatology specialist
Canakinumab	**3)** sJIA active systemic features (no active arthritis): **Yes** (Level C); **4)** sJIA, active arthritis (no active systemic features): **Yes** (Level C); **5)** sJIA with features concerning for MAS: **Unsure** (Level D)	**3)** continued disease Tx after GC monotherapy (Level A), MTX/leflunomide (Level A), anakinra (Level B), or tocilizumab (Level C); use if MD global ≥5 + any AJC even if had previous NSAID monotherapy (Level C); **4)** continued disease Tx–start if AJC >4 after trial of DMARD + anakinra/ tocilizumab (Level B), a DMARD + TNFα inhibitor (Level B), or abatacept (Level C); **5)** becomes inappropriate when MD global <5 and no prior intervention, GC monotherapy, or calcineurin monotherapy (Level D)			Not assessed	GPs to refer patient to pediatric rheumatology specialist
Calcineurin inhibitor	**3)** sJIA active systemic features (no active arthritis): **Yes** (Level C)	**3)** continued disease Tx only if MD global ≥5 + AJC = 0 after trial of IL-1 inhibitor **+** tocilizumab sequentially; inappropriate if MD global <5 + AJC = 0 (Level D), but unsure if pt tried IL-1 inhibitor + tocilizumab sequentially OR another DMARD + IL-1 inhibitor/tocilizumab			Not assessed	GPs to refer patient to pediatric rheumatology specialist
Etanercept			**Yes** (Grade A, level I)	≥ 4 years old; for polyarticular JIA	Not assessed	GPs to refer patient to pediatric rheumatology specialist
Rilanocept	**3)** sJIA active systemic features (no active arthritis): **Unsure**; **4)** sJIA, active arthritis (no active systemic features): **Unsure**; **5)** sJIA with features concerning for MAS: **Unsure**	**3)** inappropriate as initial Tx (Level D); unsure if continued DA after trying other Tx options; **4)** any AJC		Not been approved in children with JIA 12 years old??	Not assessed	GPs to refer patient to pediatric rheumatology specialist
Rituximab	**2)** Hx ≥5 joints: Yes (Level C); **3)** sJIA active systemic features (no active arthritis): **Varies**; **4)** sJIA, active arthritis (no active systemic features): **No** (Level D); **5)** sJIA with features concerning for MAS: inappropriate (Level D)	**2)** start after TNFα inhibitor and abatacept in a row when high DA OR moderate DA and FPP; may be better for RF-positive vs. negative pts (informal recommendation); **3)** inappropriate when AJC = 0 + any MD global; inappropriate when MD global <5 + AJC <4 (Level D), but unsure if pt tried both IL-1 inhibitor and tocilizumab sequentially; inappropriate if MD global <5 + AJC >4 OR MD global ≥5 + AJC >0, but unsure if pt tried IL-1 inhibitor and tocilizumab sequentially OR a DMARD + IL-1 inhibitor/tocilizumab; **4)** AJC ≤4, but unsure if pt tried IL-1 inhibitor and tocilizumab sequentially OR a DMARD + IL-1 inhibitor/tocilizumab; inappropriate if AJC >4, but unsure if pt tried both IL-1 inhibitor + tocilizumab sequentially OR a DMARD + IL-1 inhibitor, tocilizumab, a TNFα inhibitor, or abatacept			Not assessed	GPs to refer patient to pediatric rheumatology specialist
Tocilizumab	**3)** sJIA active systemic features (no active arthritis): **Yes** (varies); **4)** sJIA, active arthritis (no active systemic features): **Yes** (Level B); **5)** sJIA with features concerning for MAS: **Unsure**	**3)** continued DA after GC monotherapy (Level A), MTX/leflunomide (Level B), or anakinra (Level B) for any MD global or AJC; if MD global ≥5 + any AJC regardless if prior NSAID monotherapy (Level C); **4)** continued disease Tx when AJC >0 after anakinra OR MTX/leflunomide	**Yes** (Grade A, level II)	≥ 2 years old; for refractory sJIA	Not assessed	GPs to refer patient to pediatric rheumatology specialist
TNF alpha inhibitors	**1)** Hx ≤4 joints: **Yes** (Level C); **2)** Hx ≥5 joints: **Yes** (Level B); **3)** sJIA active systemic features (no active arthritis): not assessed in ACR 2011 –**Yes in ACR 2013** (Level C); **4)** sJIA, active arthritis (no active systemic features): **Yes** (Level B); **6)** active sacroiliac arthritis: **Yes** (Level C)	**1)** after GC injections and 3 months of max tolerated dose MTX when high DA and FPP; after GC injections + 6 months MTX when high DA and no FPP; enthesitis-related JIA pts with GC injections and sulfasalazine trial (without MTX before) and moderate/high DA; **2)** after 3 months of max tolerated dose MTX/leflunomide when moderate/high DA; after 6 months MTX/leflunomide when low DA; switch TNFα inhibitor after 4 months if moderate/high DA (Level C); switch to a TNFα inhibitor if receiving abatacept for 3 months and high DA with FPP, OR 6 months of abatacept and moderate/high DA (any FPP) (Level D); **3)** ACR 2011: not effective for Tx active systemic features; continued disease Tx–start if AJC >4 + any MD global after trial of IL-1 inhibitor/tocilizumab; if AJC >0 + any MD global after trial IL-1 inhibitor + tocilizumab sequentially; inappropriate if MD global <5 + AJC = 0 (Level D), but unsure if tried IL-1 inhibitor + tocilizumab sequentially OR DMARD + IL-1 inhibitor/tocilizumab; inappropriate if MD global ≥5 + AJC = 0 (Level D), but unsure if pt tried IL-1 inhibitor/tocilizumab; **4)** concomitant Tx of MTX 3 months when moderate/high DA; may need to switch anakinra to TNFα inhibitor when moderate/high DA–risk is latent systemic DA may be revealed (Level D); continued active Tx–start if AJC >0 after MTX/leflunomide (Level C), anakinra (Level D), or tocilizumab (Level D); **6)** after NSAIDs trial when high DA and FPP; after 3 months MTX when moderate/high DA OR moderate DA and FPP, OR after 6 months MTX when moderate DA without FPP; if 3 months sulfasalazine when moderate/high DA, OR 6 months sulfasalazine when low DA without FPP; inappropriate for any MD global (Level D), but unsure when calcineurin inhibitor + anakinra	**Yes** (Grade A, level I)	if not enough response to NSAIDs and GC injections OR if no response to MTX; for poly-JIA	Not assessed	GPs to refer patient to pediatric rheumatology specialist
**Other**						
Hydroxychloroquine monotherapy**	**1)** Hx ≤4 joints: **No** (Level C); **2)** Hx ≥5 joints: inappropriate (Level A)	**1)** inappropriate for active arthritis; with/without concurrent NSAID Tx; **2)** inappropriate to start for active arthritis; with/without concurrent NSAID Tx	Not assessed	Clinical trials revealed limited efficacy		
Autologus stem cell transplantation (SCT)			Level III	Only as a last resort because of serious adverse events		
Simple analgesics					**Yes** (Grade C)	Use paracetamol; varies by body mass; seek medical advice for use >48 hours; use as short-term Tx
Weak opioids					**Yes** (Grade D)	Codeine is weak opioid of choice; prescribe with paracetamol for moderate pain of articulations; varies by body mass; seek medical advice for use >48 hours
Intravenous immune-globulin (IVIG)	**3)** sJIA active systemic features (no active arthritis): inappropriate (Level D); **5)** sJIA with features concerning for MAS: inappropriate (Level D)	**3)** any AJC or MD global; **5)** unsure if pt tried a calcineurin inhibitor + anakinra				
Comple- mentary and alternative medicines (CAM)					**Unsure** (Grade D)	Ask pt/parents about use of CAM and possibly inform that no RCTs or systematic reviews available in children; low risk intervention, but interactions with medications a concern

DMARDs = disease-modifying antirheumatic drugs; NSAIDs = non-steroidal anti-inflammatory drugs; MTX = methotrexate; GC = glucocorticoid

Hx = history; DA = disease activity; FPP = features of poor prognosis (see ACR 2011 & 2013 for detailed description); AJC = active joint count; MD global = physician global assessment (10-point numeric rating scale); SC = subcutaneous; b.i.d. = twice daily; o.d. = once daily; pt = patient

oligo-JIA = oligoarticular JIA; poly-JIA = polyarticular JIA; sJIA = systemic JIA

**Table 4:** Summary of recommendations for non-pharmacological interventions in JIA based on included CPGs.

	GKJR [27] recommendation		RACGP [28] recommendation	
	Recommend: Yes/No/Unsure (Grade)	Details	Recommend: Yes/No/Unsure (Grade)	Details
Complementary/alternative physical therapies			**Unsure** (Grade D)	No research available using children with JIA
Electrotherapy and ultrasound	**Yes** (Grade B, level III)	For pts with tendosynovitis		
Exercise	**Yes** (Grade A, level I)	Depends on amount of inflammation, number of joints affected and global assessment of disease activity; low-impact sports preferred so easier on joints	**1)** Land-based: **Yes** (Grade C); **2)** Aquatic: **Yes** (Grade C)	**1)** regular activity according to pt’s physical abilities and any disease restrictions; **2)** recommended on case-by-case basis; is safe exercise for pts
Massage and lymph drainage	**Yes** (Grade B, level III)			
Nutritional therapy			**Yes** (Grade B)	Monitor calcium intake and provide advice about increasing intake; consider giving calcium and vitamin D supplements, especially pts on corticosteroids due to higher risk of osteoporosis and osteopenia
Orthotics management (corrective appliances/splints)	**Yes** (Grade B, level I)	For axial misalignment, prevention false weight bearing, joint stabilization; case-by-case decision by physician	**1)** Splints: **Yes** (Grade D); **2)** Foot orthotics: **Yes** (Grade D)	**1)** recommended on case-by-case basis with help of a multidisciplinary team of health professionals; includes resting + functional splints experienced fitting done by experienced therapist; **2)** recommended on case-by-case basis with help of a multidisciplinary team of health professionals; for JIA affecting the lower limbs
Physiotherapy/occupational therapy	**Yes** (Grade A, level II)	In combination with pharmacological therapy; provide pts with exercise sessions for joint mobility		
Psychological support	**Yes** (Grade A, level III)	In combination with standard pediatric rheumatology care; identify and treat mental and behavioural problems related to physical aspects of disease		
Socio-pedagogical care	**Yes** (Grade A, level III)	Includes help with school, work and daily life integration; educate parents and pts		
Synovectomy	**Yes** (Grade B, level III)	Open or arthroscopic; use on case-by-case basis only after other therapies are unsuccessful		
Thermotherapy	**Yes** (Grade A, level II)	Use cold appliances for acute joint inflammation	**Yes** (Grade D)	Use heat or cold packs, warm baths and/or ice massage; for relief of JIA symptoms

N.B. This is meant as a summary of the recommendations only. Please refer to the official clinical practice guidelines for more details on the types of interventions in broader categories (e.g. complementary and alternative physical therapies)

pt = patient

#### RACGP 2009

The RACGP CPG was focused on providing recommendations within an Australian healthcare context for general practitioners (GPs) and other multidisciplinary healthcare professionals who provide support and/or care for patients with JIA. The population of interest consisted of patients less than 16 years who have at least one joint that is painful and swollen [28]. The CPG directs GPs on early diagnosis of JIA, initial treatment options, and timing of referral to a pediatric rheumatologist or other healthcare professionals, all with a multidisciplinary focus [28]. Pharmacological interventions considered were oral NSAIDs, topical NSAIDs, simple analgesics (e.g. acetaminophen), weak opioids, and complementary and alternative medicines. DMARDs and biologics were not considered as they are beyond the scope of interventions prescribed by GPs. Thus, GPs were directed to refer patients to a pediatric rheumatologist. Non-pharmacological interventions included were complementary and alternative physical therapies (e.g. transcutaneous electrical nerve stimulation (TENS), laser therapy, acupuncture, ultrasound), exercise (land-based and aquatic), nutritional therapy, orthotics management (corrective appliances/splints), and thermotherapy [28].

Recommendations were presented through algorithms for the diagnosis, early management and long-term monitoring of JIA. Once a diagnosis of JIA has been made, it is suggested to commence initial management of disease using short term analgesics (e.g. acetaminophen) and an NSAID [28]. When symptoms of inflammation continue for more than 4 weeks, patients should be referred to a pediatric rheumatologist for advanced treatment (GC intra-articular injection, oral or intravenous corticosteroids, DMARDs, and biologics) [28]. In the 6 to 12 weeks after diagnosis, basic pharmacological treatment (simple analgesics such as acetaminophen, NSAIDs and weak opioids) can be provided by the GP, while consulting a pediatric rheumatologist [28]. Beyond 12 weeks, the GP should monitor the child or adolescent and refer them to the appropriate multidisciplinary healthcare professionals (e.g. physiotherapists, occupational therapists, dieticians, podiatrists) who can provide non-pharmacological interventions as an additional component of a patient’s care plan. GPs are also advised to manage acute flares of JIA using analgesics with or without NSAIDs and to involve a pediatric rheumatologist and other healthcare professionals in the event that disease flares continue [28].

#### GKJR 2012

The GKJR CPG was intended for a variety of individuals, including physicians and other healthcare professionals who care for patients with uncomplicated JIA [27]. Multidisciplinary treatment is recommended, including both pharmacological and non-pharmacological interventions. The types of pharmacological interventions considered were NSAIDs, GCs (intra-articular injection and systemic), DMARDs, and biologic agents (tumor necrosis factor alpha (TNFα) inhibitors, interleukin-1 (IL-1) inhibitors, interleukin-6 (IL-6) inhibitors, and co-stimulatory antagonists). Non-pharmacological interventions included physiotherapy and occupational therapy, low-impact sports and exercise, nutritional therapy, surgery, and psychological and socio-pedagogical care [27]. The pharmacological recommendations for systemic GCs, DMARDs and biologics sometimes specified if they were for oligoarticular JIA, polyarticular JIA, or sJIA; otherwise, the recommendations were general. The CPG did not discuss how to treat those individuals who were resistant to medications or obscure and difficult arthritis cases [27].

The clinical recommendations were presented for each JIA onset type. For oligoarticular JIA, treatment starts with an NSAID, then intra-articular injections (with GCs) are added and finally DMARDs (specifically methotrexate (MTX)). For polyarticular JIA, the initial treatment is a combination of an NSAID plus GCs (intra-articular injection or systemic). As an escalation of treatment, the next step is to treat with a DMARD (specifically MTX). Finally, if disease activity is high, a TNFα inhibitor should be added to the treatment, but if disease activity is low, the DMARD should be replaced with a TNFα inhibitor. For sJIA, NSAID and systemic GC (high dose or pulse) interventions are initially recommended, followed by the addition of DMARDs (specifically MTX) as an escalation in treatment. Finally, a biologic agent should be added if disease activity continues. For each onset type, physiotherapy interventions should be used concomitantly throughout the escalation of pharmacological interventions according to GKJR in order to ensure a multidisciplinary approach to care [27].

#### ACR 2011 & 2013

The ACR CPG was aimed toward pediatric rheumatologists to give recommendations on NSAIDs, GCs (intra-articular injection and systemic), DMARDs, and biologics [29,30]. The recommended pharmacological intervention depends on whether disease activity is low, moderate, or high and if an individual has features of poor prognosis or not [29,30]. The 2011 ACR CPG focused on the most frequently used medications in each drug class, while the 2013 ACR CPG update included medications specific to treating sJIA [29,30]. There was no discussion of contraindications, intolerance, tapering or discontinuing medications in the event of inactive disease [29]. Instead of presenting the patient populations of interest according to JIA onset types, ACR used the following clinical scenarios: 1) history of arthritis in 4 or fewer active joints, 2) history of arthritis in 5 or more active joints, 3) sJIA with active systemic features but no active arthritis, 4) sJIA with active arthritis but no systemic features. Other clinical scenarios considered were active sacroiliac arthritis in the 2011 CPG and sJIA with features of macrophage activation syndrome (MAS) in the 2013 update [29,30].

Features of poor prognosis varied according to clinical scenario and were clearly defined in tables in the CPG [29]. For those with a history of 4 or fewer joints at least one of the following features of poor prognosis had to be satisfied: arthritis of the hip or cervical spine, 2) arthritis of the ankle or wrist plus a noticeable or extended time with elevated inflammatory markers and 3) radiographic damage (erosion of joint or reduction in joint space) [29]. For those with a history of 5 or more joints, the features are the same except for the second one, which is positive rheumatoid factor or presence of anticyclic citrullinated peptide antibodies [29]. The clinical scenario of systemic arthritis with active arthritis but without active systemic features has only two features of poor prognosis: arthritis of the hip and radiographic damage (erosion of joint or reduction in joint space) [29]. For sJIA with active systemic features but without active arthritis the features of poor prognosis were defined as: 1) systemic disease being active for at least 6 months (fever, elevated inflammatory markers), or 2) need for systemic GC intervention [29]. The 2013 update used active joint count and physician’s global assessment to assist in determining the need for specific interventions rather than the features of poor prognosis [30].

Each clinical scenario has described disease activity levels being low, moderate, or high. There were specific requirements for each scenario, but common among them for low disease activity was having a normal erythrocyte sedimentation rate or C-reactive protein level and receiving a score below 2/10 on the patient/parent global assessment of overall well-being [29]. For moderate disease activity, each clinical scenario required that patients experience one or more low disease activity criterion, but under three that were considered high disease activity [29]. To be classified as high disease activity, among other specific criteria of each clinical scenario, patients must have a doubled erythrocyte sedimentation rate or C-reactive protein level compared to what is considered the upper limit of normal and have a score of 7 or higher out of 10 on the physician global assessment of overall disease activity. Unlike with low disease activity where all criteria must be satisfied, all but one of the criteria for a particular clinical scenario could be satisfied. Systemic arthritis with active systemic features but without active arthritis had two levels of disease activity, depending on the physician global assessment of overall disease activity (above or below 7/10) [29].

The ACR CPGs [29,30] made separate treatment algorithms for each of their clinical scenarios. For a history of 4 or fewer joints, NSAID monotherapy is given initially to those with low disease activity without features of poor prognosis (FPP) and to start on GC injections after 2 months if the disease activity (DA) remains or worsens. For any other combination of features of poor prognosis (FPP) and disease activity (DA), the initial treatment is GC intra-articular injections with NSAIDs as necessary. The escalation of treatment would lead to MTX as the next step for any of these patients (with NSAIDs or GC intra-articular injections as necessary) and then finally a TNFα inhibitor (with NSAIDs or GC intra-articular injections as necessary). Among individuals with a history of 5 or more joints, MTX is recommended (with NSAIDs or GC intra-articular injections as necessary) when there is high disease activity (DA) or moderate disease activity with features of poor prognosis or after 1 month of NSAIDs for low disease activity and features of poor prognosis or moderate disease activity and any prognosis [29]. After MTX treatment for either 3 or 6 months, depending on disease activity, a TNFα inhibitor (with NSAIDs or GC injections as necessary) should be given. Finally, if after 4 months the disease activity is still moderate or high then a different TNFα inhibitor or abatacept should also be prescribed in the place of the first one [29]. For systemic arthritis with active systemic features, NSAIDs should be the initial intervention only if the physician’s global assessment is less than 7 and the patient presents with fever, but has no features of poor prognosis. Otherwise they should be started on systemic GCs (with NSAIDs as necessary). If fever continues, the treatment is escalated to anakinra (with GC intra-articular injections or NSAIDs as necessary). The treatment pathways are more complex in the 2013 update and the pathways depend on the active joint count for determining the disease activity. With a lower joint count (no more than 4) initial intervention will be either NSAID monotherapy or GC intra-articular injections, but MTX or leflunomide will be the initial intervention when more than 4 joints have active disease [30].

## Discussion

Of the included CPGs for this critical appraisal, RACGP [28] and ACR [29,30] were found to be of high quality and were recommended with or without modifications by all three appraisers, but GKJR [27] was considered of lower quality. Clinicians must therefore use their discretion in applying the recommendations from GKJR. Domains 1–4 generally had high scores for all three CPGs; they concern scope and purpose, stakeholder involvement, rigor of development, and clarity of presentation, respectfully. On the other hand, domains 5 and 6, which address applicability and editorial independence, received the lowest scores as they were not well addressed in any of the CPGs. The differences in the CPGs in terms of context, target audience, patient population and clinical scenarios made it challenging to compare the recommended interventions. Furthermore, each CPG provided their own criteria for grading (i.e. strength of recommendations and level of evidence) and used different scales (e.g. a 3-level or 5-level grading scale).

### Assessment of the quality of the CPGs

The elevated mean score for scope and purpose (domain 1) was similar to what has been found for reviews in rheumatology and various pediatric conditions [19,20,23,31,32].

RACGP [28] scored the highest for stakeholder involvement (domain 2) because it provided more clear information about the CPG development group, the input of the target population, and the CPG’s target users. Interestingly, this domain saw high scores for the CPGs, while CPGs in adult rheumatology had lower scores [23,31,33], but high scores were seen in a review of CPGs in pediatrics [32].

For rigor of development (domain 3), a lower quality score was given to ACR [29,30] and GKJR [27] for not providing clear strengths and limitations to the body of evidence (item 9). Overall, RACGP [28] received a high quality score for receiving fairly high scores in all items except 13 and 14. A similar review examining osteoarthritis also found scores for items 9 and 14 to be lower [19].

Previous reviews of CPGs in rheumatology and various pediatric conditions also gave high quality scores for clarity of presentation (domain 4), whereas applicability (domain 5) consistently received the lowest scores of all domains [19,20,23,31–33]. A great deal of time and resources are required to develop a CPG and they should be reassessed every 3–5 years for validity in order to remain up to date. The developers of the CPGs likely do not see it as feasible to focus as much on applicability as other areas of CPG quality because it may involve monitoring or auditing of the recommendations being implemented [28,34].

For the most part, the low scores for editorial independence (domain 6) were also consistent with previous reviews in rheumatology [19,20,23,31–33].

### Implications of the quality scores

Both ACR [29,30] and RACGP [28] were of higher methodological quality after assessment using the AGREE II instrument and thus clinicians can trust the methodological quality of these CPGs for use in practice [21]. RACGP [28] and GKJR [27] were specific in reporting pharmacological interventions, but the quality and specificity of the reporting on non-pharmacological interventions was lacking. For example, both CPGs covering the non-pharmacological management of JIA recommended exercise, which is far too broad of a category as it could include resistance, aerobic, strengthening and other forms of exercise. Certain types of exercise would be more beneficial while others may be more harmful for patients with JIA. A similar problem was found when GKJR [27] referred to physical and occupational therapy as interventions when those are professions.

Interestingly, RACGP [28] used the AGREE instrument as a guide for developing their CPG. Of the included CPGs it received the highest overall quality score by all three appraisers for this review. Certain recommendations in RACGP [28] for both pharmacological and non-pharmacological interventions had lower grades because the CPGs used research on adults with osteoarthritis or rheumatoid arthritis and extrapolated to the target population of patients with JIA [28]. This led to lower recommendation grades, because of a lack of high quality studies available. A systematic review on intra-articular steroid injections for managing JIA indicated a lack of RCTs using children with JIA and that any extrapolations from adult studies must be done with care [35].

### Recommended interventions

Due to the differences across CPGs in terms of context, target audience, and clinical scenarios considered, the kinds of recommendations provided did not always overlap, so recommendations were summarized separately by CPG. For example, ACR provides recommendations on pharmacological interventions specific for pediatric rheumatologists (e.g. DMARDs, biologics), whereas RACGP had GPs as the target audience, thus the pharmacological interventions considered for recommendation were limited to initial interventions for low disease activity [28–30]. This was also observed for a critical appraisal of CPGs for osteoarthritis in the knee, with some providing very specific recommendations and others being more general [23]. Another critical appraisal of CPGs for osteoarthritis found that combining recommendations from multiple CPGs was not possible due to elevated levels of variation [31].

Among newly diagnosed patients, the RACGP CPG recommended that GPs provide NSAIDs to reduce inflammation and pain [28]. As disease severity increases or after 4 weeks from onset of symptoms the role of the GP is to refer the patient to a pediatric rheumatologist for specific prescriptions of other pharmacological interventions [28]. Non-pharmacological interventions should be implemented from the start of management of the disease from the appropriate healthcare professionals based on the RACGP experts’ opinion [28].

Both ACR and GKJR provided recommendations for longer term and with specific clinical scenarios in mind. The ACR clinical scenario of 4 or fewer active joints includes oligoarthritis, which was one of the onset types with specific recommendations provided by GKJR. The initial treatment of patients within this clinical scenario was similar in all CPGs, with NSAIDs being recommended initially (for low disease activity but no features of poor prognosis in ACR). The progression of treatment was similar between the GKJR and the ACR CPGs, which includes the addition of GC intra-articular injections, and MTX as the next intervention [27,29]. Only ACR recommended to switch to a TNFα inhibitor with NSAIDs and/or GC intra-articular injections as required after either 3 or 6 months of MTX, depending on disease activity for patients with a history of 4 or fewer active joints (which includes oligoarthritis in the GKJR CPG) [29]. GKJR considered non-pharmacological interventions as well as pharmacological interventions and suggested that throughout the progression of treatment, this patient population should receive physiotherapy interventions [27]. ACR provided more detailed information on pharmacological interventions as it considered an individual’s level of disease activity and the presence or absence of features of poor prognosis [29]. In contrast, GKJR did not mention features of poor prognosis in its recommendations and was inconsistent in providing details on disease activity. For example, the recommendations for MTX and systemic GCs included patients with highly active JIA and one of the three algorithms differentiated the pathway for low and high disease activity, but the consensus statements for other pharmacological interventions did not provide details on the level of disease activity [27].

The clinical scenario in ACR of a history of 5 or more active joints included polyarticular JIA, as described in the GKJR CPG. While more details for the specific context (e.g. disease severity and presence of features of poor prognosis) were provided by ACR, both CPGs indicated a similar progression of treatment. The main differences were that for GKJR, a TNFα inhibitor was to be added to the DMARD (preferably MTX) for high disease activity rather than replacing the DMARD with a TNFα inhibitor as with low disease activity, and ACR recommends switching to a different TNFα inhibitor or abatacept after 4 months if disease activity is moderate to high [27,29]. Physiotherapy interventions were the non-pharmacological interventions recommended for the entire progression of treatment in GKJR [27,29].

In the ACR CPG, the algorithm for sJIA with active systemic features but no active arthritis focused on interventions to target the systemic symptoms starting with systemic GCs and then anakinra later on. This specific type of sJIA was not considered in the GKJR CPG [27,29]. The algorithm for treating sJIA in GKJR was similar to the clinical scenario presented by ACR for sJIA with active arthritis. Following treatment with an NSAID and GC intra-articular injections, MTX is the recommended DMARD to add to the treatment. Next, GKJR recommended adding a biologic, but ACR provides two possible interchangeable pathways: anakinra or a TNFα inhibitor and then abatacept if disease activity is moderate to high after 4 months [27,29].

Both the RACGP and GKJR [27] CPGs described non-pharmacological interventions, but there was not always a clear description of the disease severity and stage of disease (e.g. acute versus chronic). For GKJR, no specific details were provided for the broad disciplines of physiotherapy and occupational therapy, which were listed as interventions [27,28]. In addition, the recommendation grades for exercise were different between RACGP and GKJR, since GKJR [27] combined all evidence for exercise into one broad category while RACGP [28] separated land-based and aquatic exercises. A Cochrane systematic review published in 2008 based on three RCTs on exercise concluded that there were no statistically significant or clinically important improvements for clinical outcomes with exercise interventions [36], yet GKJR was published more recently and indicates there is high quality evidence to support a high recommendation for exercise.

### Limitations

The AGREE II instrument has undergone some revisions since the development of the original AGREE instrument. One change was to use a 7-point scale for evaluating the items in the domains instead of a 4-point scale. This may have been a limitation in assessing the quality of the CPGs because the only well-defined points in the scale are 1 and 7 [21]. There is little to distinguish the values 3, 4, and 5, thus it may have introduced a potential risk of reporting bias.

In addition, the AGREE II instrument is limited in terms of the information that is in focus for scoring the CPGs on the items in each domain. For example, GKJR [27] and RACGP [28] were both providing recommendations on pharmacological and non-pharmacological interventions, but there is no distinction in the AGREE II quality assessment for different intervention types, so appraisers had provided scores for each item for the CPG in its entirety, rather than on specific sections (i.e. pharmacological and then non-pharmacological intervention recommendations).

There was also a risk of selection bias because only studies published in English or French were included. Finally, due to the low number of included CPGs, it was not possible to calculate the intra-class correlation coefficient to determine the inter-rater reliability as the results would not converge. The test was not sensitive enough to detect such a low number of items and the assumptions of the reliability model were violated. However, by resolving the two medium discrepancies found in the concordance calculator and by assessing the raw data scores, the three appraisers maintained fairly consistent scores.

## Conclusions

The ACR [29,30] and RACGP [28] CPGs were found to be of higher quality using the AGREE II instrument while the GKJR [27] CPG was considered to be of lower quality. Future CPGs should focus on improving the methodological quality, particularly for monitoring the implementation of recommendations and assessing the CPG’s impact. Recommendations varied across the CPGs due to differences in context, target audience and patients’ clinical presentation of disease. Even though there was variability in CPGs, the progression of pharmacological treatment did not seem to be in conflict between CPGs. RACGP and GKJR covered different non-pharmacological interventions and the recommendations were vague. Overall, both pharmacological and non-pharmacological recommendations were based on a paucity of evidence and studies of weaker design. Therefore, there is a need for further research on interventions for JIA in order to have higher quality evidence to ensure greater confidence for clinicians to implement the recommendations of a particular CPG in rheumatology practice.

## Supporting Information

S1 Appendix PRISMA Checklist. (DOC)

S2 Appendix Literature search strategies. (DOC)

S3 Appendix Selection criteria of the clinical practice guidelines. (DOC)

S4 Appendix Reasons for excluding certain citations. (DOC)

S5 Appendix Grading systems for recommendations of included clinical practice guidelines. (DOCX)

S1 Fig PRISMA flow diagram. (DOC)

## References

[1] Manners PJ, Bower C. Worldwide prevalence of juvenile arthritis why does it vary so much? J Rheumatol. 2002;29: 1520–1530. 12136914

[2] Stanley LC, Ward-Smith P. The diagnosis and management of juvenile idiopathic arthritis. J Pediatr Health Care. 2011;25: 191–194. 10.1016/j.pedhc.2010.12.003 21514495

[3] Petty RE, Southwood TR, Manners P, Baum J, Glass DN, Goldenberg J, et al International League of Associations for Rheumatology classification of juvenile idiopathic arthritis: second revision, Edmonton, 2001. J Rheumatol. 2004;31: 390–392. 14760812

[4] Petty RE, Laxer RM, Wedderburn LR. Juvenile idiopathic arthritis In: Laxer RM, Lindsley CB, Wedderburn LR, editor. Textbook of pediatric rheumatology. Philadelphia, PA: Elsevier; 2016 pp. 188–204.

[5] Johnson CJ, Reilly KM, Murray KM. Etanercept in juvenile rheumatoid arthritis. Ann Pharmacother. 2001;35: 464–471. 11302411 10.1345/aph.10123

[6] Bromberg MH, Connelly M, Anthony KK, Gil KM, Schanberg LE. Self-reported pain and disease symptoms persist in juvenile idiopathic arthritis despite treatment advances: an electronic diary study. Arthritis Rheumatol. 2014;66: 462–469. 10.1002/art.38223 24504820 PMC4172333

[7] Schanberg LE, Anthony KK, Gil KM, Maurin EC. Daily pain and symptoms in children with polyarticular arthritis. Arthritis Rheum. 2003;48: 1390–1397. 12746912 10.1002/art.10986

[8] Bloom BJ, Owens JA, McGuinn M, Nobile C, Schaeffer L, Alario AJ. Sleep and its relationship to pain, dysfunction, and disease activity in juvenile rheumatoid arthritis. J Rheumatol. 2002;29: 169–173. 11824956

[9] Tupper SM, Rosenberg AM, Pahwa P, Stinson JN. Pain intensity variability and its relationship with quality of life in youths with juvenile idiopathic arthritis. Arthritis Care Res (Hoboken). 2013;65: 563–570. 22972759 10.1002/acr.21850

[10] Hashkes PJ. Pediatric rheumatology: Strengths and challenges of a new guide for treating JIA. Nat Rev Rheumatol. 2011;7: 377–378. 10.1038/nrrheum.2011.67 21587238

[11] Hayward K, Wallace CA. Recent developments in anti-rheumatic drugs in pediatrics: treatment of juvenile idiopathic arthritis. Arthritis Res Ther. 2009;11: 216 10.1186/ar2619 19291269 PMC2688259

[12] Boros C, Whitehead B. Juvenile idiopathic arthritis. Aust Fam Physician. 2010;39: 630–636. 20877765

[13] Cavallo S, April KT, Grandpierre V, Majnemer A, Feldman DE. Leisure in children and adolescents with juvenile idiopathic arthritis: a systematic review. PLoS One. 2014;9: e104642 10.1371/journal.pone.0104642 25329390 PMC4203655

[14] Weiss JE, Luca NJ, Boneparth A, Stinson J. Assessment and management of pain in juvenile idiopathic arthritis. Paediatr Drugs. 2014;16: 473–481. 10.1007/s40272-014-0094-0 25331986

[15] Davies K, Cleary G, Foster H, Hutchinson E, Baildam E, British Society of Paediatric and Adolescent Rheumatology. BSPAR Standards of Care for children and young people with juvenile idiopathic arthritis. Rheumatology (Oxford). 2010;49: 1406–1408. 20173199 10.1093/rheumatology/kep460

[16] Field MJ Lohr KN. Clinical practice guidelines: Directions for a new program Washington, DC: National Academy Press; 1990. 25144032

[17] Brouwers MC, Kho ME, Browman GP, Burgers JS, Cluzeau F, Feder G, et al Development of the AGREE II, part 2: assessment of validity of items and tools to support application. CMAJ. 2010;182: E472–8. 10.1503/cmaj.091716 20513779 PMC2900368

[18] Higgins JPT, Green S (editors). Cochrane handbook for systematic reviews of interventions version 5.1.0 Cochrane Collaboration 2011 Available: www.cochrane-handbook.org.

[19] Brosseau L, Rahman P, Toupin-April K, Poitras S, King J, De Angelis G, et al A Systematic Critical Appraisal for Non-Pharmacological Management of Osteoarthritis Using the Appraisal of Guidelines Research and Evaluation II Instrument. PLoS One. 2014;9: e82986 10.1371/journal.pone.0082986 24427268 PMC3888378

[20] Brosseau L, Rahman P, Poitras S, Toupin-April K, Paterson G, Smith C, et al A systematic critical appraisal of non-pharmacological management of rheumatoid arthritis with Appraisal of Guidelines for Research and Evaluation II. PLoS One. 2014;9: e95369 10.1371/journal.pone.0095369 24840205 PMC4026323

[21] Brouwers MC, Kho ME, Browman GP, Burgers JS, Cluzeau F, Feder G, et al AGREE II: advancing guideline development, reporting, and evaluation in health care. Prev Med. 2010;51: 421–424. 10.1016/j.ypmed.2010.08.005 20728466

[22] Levinson AJ, Garside S, Bousfield J, Bousfield J, Large C, Levesque M, et al. AGREE II Overview tutorial. Available: http://www.agreetrust.org/resource-centre/agree-ii-training-tools/.

[23] Poitras S, Avouac J, Rossignol M, Avouac B, Cedraschi C, Nordin M, et al A critical appraisal of guidelines for the management of knee osteoarthritis using Appraisal of Guidelines Research and Evaluation criteria. Arthritis Res Ther. 2007;9: R126 18062805 10.1186/ar2339 PMC2246248

[24] Yan J, Min J, Zhou B. Diagnosis of pheochromocytoma: a clinical practice guideline appraisal using AGREE II instrument. J Eval Clin Pract. 2013;19: 626–632. 10.1111/j.1365-2753.2012.01873.x 22809219

[25] Hurkmans EJ, Jones A, Li LC, Vliet Vlieland TP. Quality appraisal of clinical practice guidelines on the use of physiotherapy in rheumatoid arthritis: a systematic review. Rheumatology (Oxford). 2011;50: 1879–1888. 21743086 10.1093/rheumatology/ker195

[26] McMaster University. AGREE II rater concordance calculator. 2008. Available: http://fhswedge.csu.mcmaster.ca/cepftp/qasite/AGREEIIRaterConcordanceCalculator.html.

[27] Dueckers G, Guellac N, Arbogast M, Dannecker G, Foeldvari I, Frosch M, et al Evidence and consensus based GKJR guidelines for the treatment of juvenile idiopathic arthritis. Clin Immunol. 2012;142: 176–193. 10.1016/j.clim.2011.10.003 22154868

[28] Munro J, Brun S, Rawlin M, Webster P, Grimmer-Somers K, Jasper A, Rada J, Haesler E. Evidence and consensus based GKJR guidelines for the treatment of juvenile idiopathic arthritis South Melbourne, Victoria: Royal Australian College of General Practitioners; 2009.

[29] Beukelman T, Patkar NM, Saag KG, Tolleson-Rinehart S, Cron RQ, DeWitt EM, et al 2011 American College of Rheumatology recommendations for the treatment of juvenile idiopathic arthritis: initiation and safety monitoring of therapeutic agents for the treatment of arthritis and systemic features. Arthritis Care Res (Hoboken). 2011;63: 465–482. 21452260 10.1002/acr.20460 PMC3222233

[30] Ringold S, Weiss PF, Beukelman T, DeWitt EM, Ilowite NT, Kimura Y, et al 2013 update of the 2011 American College of Rheumatology recommendations for the treatment of juvenile idiopathic arthritis: recommendations for the medical therapy of children with systemic juvenile idiopathic arthritis and tuberculosis screening among children receiving biologic medications. Arthritis Rheum. 2013;65: 2499–2512. 10.1002/art.38092 24092554 PMC5408575

[31] Larmer PJ, Reay ND, Aubert ER, Kersten P. Systematic review of guidelines for the physical management of osteoarthritis. Arch Phys Med Rehabil. 2014;95: 375–389. 10.1016/j.apmr.2013.10.011 24184307

[32] Sabharwal S, Patel NK, Gauher S, Holloway I, Athanasiou T. High methodologic quality but poor applicability: assessment of the AAOS guidelines using the AGREE II instrument. Clin Orthop Relat Res. 2014;472: 1982–1988. 10.1007/s11999-014-3530-0 24566890 PMC4016437

[33] Alonso-Coello P, Irfan A, Sola I, Gich I, Delgado-Noguera M, Rigau D, et al The quality of clinical practice guidelines over the last two decades: a systematic review of guideline appraisal studies. Qual Saf Health Care. 2010;19: e58 10.1136/qshc.2010.042077 21127089

[34] Shekelle PG, Ortiz E, Rhodes S, Morton SC, Eccles MP, Grimshaw JM, et al Validity of the Agency for Healthcare Research and Quality clinical practice guidelines: how quickly do guidelines become outdated? JAMA. 2001;286: 1461–1467. 11572738 10.1001/jama.286.12.1461

[35] Wallen M, Gillies D. Intra-articular steroids and splints/rest for children with juvenile idiopathic arthritis and adults with rheumatoid arthritis. Cochrane Database Syst Rev. 2006;1: CD002824 16437446 10.1002/14651858.CD002824.pub2 PMC8453330

[36] Takken T, Van Brussel M, Engelbert RH, Van Der Net J, Kuis W, Helders PJ. Exercise therapy in juvenile idiopathic arthritis: a Cochrane Review. Eur J Phys Rehabil Med. 2008;44: 287–297. 18762738

