# Supplementary material for: A Systematic Critical Appraisal of Clinical Practice Guidelines in Juvenile Idiopathic Arthritis Using the Appraisal of Guidelines for Research and Evaluation II (AGREE II) Instrument
Source: PLoS One. 2015 Sep 10;10(9):e0137180. doi: 10.1371/journal.pone.0137180 (PMC4565560; doi:10.1371/journal.pone.0137180)
Supplement: S2 Appendix — (DOC) [file pone.0137180.s002.doc]

**Appendix S2.** Literature search strategies.

**Embase**

1 arthritis, juvenile rheumatoid.mp. [mp=title, abstract, subject headings, heading word, drug trade name, original title, device manufacturer, drug manufacturer, device trade name, keyword]

2 limit 1 to "all child (0 to 18 years)" [Limit not valid in Embase; records were retained]

3 limit 2 to English language

4 limit 3 to yr="2003 -Current"

**Medline (Ovid)**

1 Arthritis, Juvenile Rheumatoid/

2 limit 1 to (english and guideline)

3 limit 2 to (english language and yr="2003 -Current" and guideline)

**Cumulative Index to Nursing and Allied Health Literature (CINAHL)**

1 (MH "Arthritis, Juvenile Rheumatoid"); Expanders - Apply related words; Search modes - Boolean/Phrase

2 (MH "Arthritis, Juvenile Rheumatoid"); **Limiters** – Published Date: 2003/01/01-2014/02/01; English Language; Peer Reviewed; Publication Type: Practice Guidelines, Systematic Review; **Expanders** - Apply related words; **Search modes** - Boolean/Phrase

**PubMed**

1 juvenile rheumatoid arthritis

2 juvenile rheumatoid arthritis Filters: Guideline

3 juvenile rheumatoid arthritis Filters: Guideline; Practice Guideline

4 juvenile rheumatoid arthritis Filters: Guideline; Practice Guideline; Publication date from 2003/01/01 to 2014/02/01

5 juvenile rheumatoid arthritis Filters: Guideline; Practice Guideline; Publication date from 2003/01/01 to 2014/02/01; English

**Canadian Medical Association (CMA) Infobase**

1 Juvenile rheumatoid arthritis

2 limit 1 to English language; Population: adolescent, child, infant; Published Date: 2003/01/01-2014/02/01

**Physiotherapy Evidence Database (PEDro)**

1 Juvenile rheumatoid arthritis; Method: practice guideline

**National Guidelines Clearinghouse**

1 Juvenile rheumatoid arthritis
