# Supplementary material for: A Systematic Critical Appraisal of Clinical Practice Guidelines in Juvenile Idiopathic Arthritis Using the Appraisal of Guidelines for Research and Evaluation II (AGREE II) Instrument
Source: PLoS One. 2015 Sep 10;10(9):e0137180. doi: 10.1371/journal.pone.0137180 (PMC4565560; doi:10.1371/journal.pone.0137180)
Supplement: S3 Appendix — (DOC) [file pone.0137180.s003.doc]

**Appendix S3. Selection criteria of the clinical practice guidelines.**

| Inclusion Criteria | Rationale for the Inclusion and Exclusion Criteria | Exclusion Criteria |
| --- | --- | --- |
| 1. The CPGs must be peer reviewed | 1. Non peer reviewed CPGs are not necessarily reliable | 1. CPGs that are not peer reviewed |
| 1. The CPGs must identify the authors | 1. The author’s name must be clearly indicated for each CPG | 1. CPGs that are authored as a professional group/association |
| 1. CPGs must include a reference list | 1. The CPG must be based on scientific data and thus provide references | 1. CPGs that exclude reference list |
| 1. The CPG must use a grading system to evaluate the level of evidence for each recommendation | 1. The recommendations for the CPG should be issued on the basis of the body of evidence relevant to the research question | 1. CPGs that provide recommendations without a grading system |
| 1. CPGs clearly based on systematic reviews | 1. Systematic reviews offered a less biased recommendation (avoiding potential publication biases) | 1. CPGs not clearly based on systematic reviews |
| 1. CPGs must produce original recommendations | 1. CPGs offered original recommendations somewhat different than existing CPGs | 1. The CPGs cannot be based only on recommendations from other guideline(s) |
| 1. The CPGs must be written in English | 1. The CPGs must be written in English for the comprehension of the guidelines and AGREE II content | 1. CPGs written in languages other than English were excluded |
| 1. The CPGs must be published within years 2003 and 2014 and must mention a release date | 1. Only recent CPGs were included. The presence of release date was necessary to ensure currency | 1. CPGs published before 2003 |
| 1. CPGs must involve studies with participants ≤18 years of age | 1. Paediatrics needs more specific recommendations for rheumatology | 1. CPGs with participants >18 years of age |
| 1. CPGs must be relevant to juvenile idiopathic arthritis (JIA) in the extremities of the body | 1. JIA can lead to other complications and certain diseases have similar signs and symptoms as JIA | 1. CPGs that looked at other juvenile arthritic conditions or complications (e.g., uveitis) |
| 1. CPG must be the most up-to-date version of the guideline developed by the same research group | 1. Selecting only the newest version will prevent repetition and will account for new recommendations based on the most recent research available underlying primary articles | 1. Older CPGs may use outdated interventions for its recommendations |
