# Supplementary material for: A Systematic Critical Appraisal of Clinical Practice Guidelines in Juvenile Idiopathic Arthritis Using the Appraisal of Guidelines for Research and Evaluation II (AGREE II) Instrument
Source: PLoS One. 2015 Sep 10;10(9):e0137180. doi: 10.1371/journal.pone.0137180 (PMC4565560; doi:10.1371/journal.pone.0137180)
Supplement: S4 Appendix — (DOC) [file pone.0137180.s004.doc]

**Appendix S4.** Reasons for excluding certain citations

| Excluded publications for JIA | Reasons for Exclusion |
| --- | --- |
| Taylor et al. [2012] | Incomplete CPG that deals with screening for uveitis associated with juvenile idiopathic arthritis |
| Guellac & Niehues [2011] | Not published in English (written in German) |
| Bishop et al. [2008] | Focused on X-ray interventions for diseases with increased risk of fractures |
| Philpott et al. [2001] | Does not use a grading system to evaluate the level of evidence for each recommendation |
| Hull et al. [2001] | Published before 2003 (in 2001) and excludes a reference list |
| Heiligenhaus et al. [2012] | Not directly relevant to JIA; focuses on uveitis (a complication of juvenile arthritis). |
| Santos et al. [2012] | Does not include a grading system to evaluate the level of evidence for each recommendation |
