# Supplementary material for: A Systematic Critical Appraisal of Clinical Practice Guidelines in Juvenile Idiopathic Arthritis Using the Appraisal of Guidelines for Research and Evaluation II (AGREE II) Instrument
Source: PLoS One. 2015 Sep 10;10(9):e0137180. doi: 10.1371/journal.pone.0137180 (PMC4565560; doi:10.1371/journal.pone.0137180)
Supplement: S5 Appendix — (DOCX) [file pone.0137180.s005.docx]

**Appendix S5.** Grading systems for recommendations of included clinical practice guidelines.

| ACR 2011 & 2013^a^ | |
| --- | --- |
| **Evidence level** | **Definition** |
| A | Evidence support from randomized clinical trials |
| B | Evidence support from nonrandomized controlled studies (cohort, case-control) or randomized clinical trial extrapolations |
| C | Evidence support from uncontrolled studies (case series) or nonrandomized controlled study extrapolations or notable randomized clinical trials extrapolations |
| D | Evidence support from expert opinion not supported by published evidence |
| GKJR 2012^b^ | |
| **Evidence level** | **Definition** |
| I | Evidence from one or more well-designed randomized controlled trials |
| II | Evidence from well-designed cohort or nonrandomized controlled trials or well-designed case-control analytic studies (preference for having more than one center/research group or multiple time series with/without intervention). May also include dramatic results in uncontrolled trials. |
| III | Evidence from expert opinion based on clinical experience, descriptive studies, or of reports of expert committees |
| **Recommendation grade** | **Definition** |
| A | Good evidence supporting the recommendation that the intervention be performed |
| B | Fair evidence supporting the recommendation that the intervention be performed |
| C | Poor evidence with regards to the value or harm of the intervention; recommendations may be made on other grounds |
| D | Fair evidence to support the recommendation that the intervention **not** be performed |
| E | There is good evidence supporting the recommendation that the intervention **not** be performed |
| RACGP 2009^c^ | |
| **Recommendation grade** | **Definition** |
| A | Excellent evidence that can be trusted in guiding practice |
| B | Good evidence that the body of evidence can be trusted in guiding practice in most situations |
| C | Some evidence that the body of evidence provides some support for recommendation(s); application must be made with care |
| D | Weak evidence that the body of evidence is weak and caution must be taken in applying the recommendation |

^a^Adapted from: Beukelman T, Patkar NM, Saag KG, Tolleson-Rinehart S, Cron RQ, et al. (2011) 2011 American college of rheumatology recommendations for the treatment of juvenile idiopathic arthritis: initiation and safety monitoring of therapeutic agents for the treatment of arthritis and systemic features. Arth Care & Res 63: 465-482.
^b^Adapted from: Dueckers G, Guellac N, Arbogast M, Dannecker G, Foeldvari I, et al. (2012) Evidence and consensus based GKJR guidelines for the treatment of juvenile idiopathic arthritis. Clin Immu 142: 176-93.
^c^Adapted from: Munro J, Brun S, Rawlin M, Webster P, Grimmer-Somers K, et al. (2009) Clinical guideline for the diagnosis and management of juvenile idiopathic arthritis. South Melbourne, Victoria: Royal Australian College of General Practitioners. 38p.
