# Supplementary material for: A Systematic Critical Appraisal of Clinical Practice Guidelines in Juvenile Idiopathic Arthritis Using the Appraisal of Guidelines for Research and Evaluation II (AGREE II) Instrument
Source: PLoS One. 2015 Sep 10;10(9):e0137180. doi: 10.1371/journal.pone.0137180 (PMC4565560; doi:10.1371/journal.pone.0137180)
Supplement: S1 Fig — (DOC) [file pone.0137180.s006.doc]

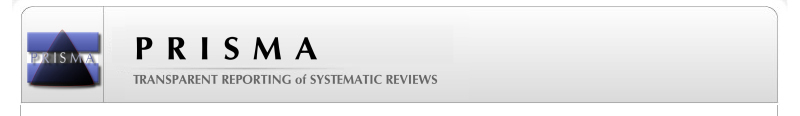
**Figure S1. PRISMA 2009 Flow Diagram.**

**Screening**

**Included**

**Eligibility**

**Identification**

Records identified through database searching
(n = 90)

Additional records identified through other sources
(n = 1)

Records after duplicates removed
(n = 88)

Records screened
(n = 39)

Records excluded
(n = 32)

Full-text articles assessed for eligibility
(n = 11)

Full-text articles excluded, with reasons
(n = 8)

- Did not have JIA interventions as its primary focus (n= 3)
- Was not published in English (n= 1)
- Did not include a grading system for evaluating the evidence level of evidence of recommendations (n= 2)
- Was published before 2003 and had no reference list (n= 1)
- Was not a true guideline (n= 1)

Studies included in qualitative synthesis
(n = 3*)

Studies included in quantitative synthesis (meta-analysis)
(n = 3*)

*N.B.: ACR (2011 & 2013) are counted as one CPG since the more recent version was a partial update.
